# Qualitative assessment of the patient experience of primary hyperoxaluria type 1: an observational study

**DOI:** 10.1186/s12882-023-03365-1

**Published:** 2023-10-26

**Authors:** David Danese, Diana Goss, Carla Romano, Catherine Gupta

**Affiliations:** 1 Alnylam Pharmaceuticals, 675 West Kendall St, Cambridge, MA 02142 USA; 2 Research Triangle Institute, Research Triangle Park, NC USA

**Keywords:** Quality of Life, SARS-CoV-2, Hyperoxaluria, Primary, Caregivers, Kidney Failure, Chronic, Kidney Stones, Qualitative Research, Oxalosis

## Abstract

**Background:**

Without effective intervention, primary hyperoxaluria type 1 (PH1) causes oxalate-induced kidney damage, leading to end-stage kidney disease and serious complications throughout the body. Although PH1 carries a heavy burden that impacts quality of life, literature on the experiences of those living with PH1 and caring for patients with PH1 is limited. This study aimed to describe the diagnostic journey in PH1 and characterize patients’ and caregivers’ self-reported experiences throughout the disease course.

**Methods:**

This was an observational study involving in-depth, semi-structured telephone interviews. Dominant trends were assessed using constant comparative analysis to identify themes in interviewees’ descriptions of their experiences. Individuals aged ≥ 12 years and caregivers of children aged 6–17 years with genetically confirmed PH1 were eligible. Informed consent/assent and ability to read and speak English were required.

**Results:**

Interviewees (16 patients, 12 caregivers) reported a prolonged diagnostic journey due to low disease awareness, among other factors. Upon diagnosis, PH1 was frequently symptomatic, typically involving kidney stone-related symptoms but also potentially symptoms arising beyond the kidneys. PH1 most commonly led to worry and social impairment in adolescents, impaired physical function in adults, and a range of impacts on caregivers. In late-stage disease, dialysis was the most burdensome aspect of living with PH1 (due to time requirements, limitations from living with a catheter, etc.), and this burden was exacerbated by the COVID-19 pandemic. Benefits desired from PH1 management included reductions in laboratory measures of oxalate burden, kidney stone and urination frequency, and oxalate-related skin ulcers.

**Conclusions:**

PH1 greatly impacts patients’ and caregivers’ lives, primarily due to burdensome disease manifestations and associated emotional, physical, and practical impacts, as well as disease management challenges – particularly those related to dialysis in late-stage disease.

## Background

Primary hyperoxalurias (PH) are a group of rare autosomal recessive genetic diseases characterized by oxalate overproduction in the liver [1–3]. Because the body relies on the kidneys to eliminate oxalate produced by the liver, PH results in excretion of excess oxalate by the kidneys, leading to formation of toxic calcium oxalate crystals in the kidneys and urinary tract and, consequently, development of chronic kidney disease (CKD) and calcium oxalate kidney stones [1, 2]. There are 3 known types of PH: PH1, PH2, and PH3 [1, 2]. PH1 is the most common (~ 80% of all PH) and severe type [1–4]. In patients with PH1 who do not receive effective intervention, oxalate-induced kidney damage almost inevitably leads to end-stage kidney disease (ESKD) [1, 5]. Kidney impairment and consequent decreases in urine output, resulting in incomplete clearance of oxalate from the body by the kidneys, can in turn lead to accumulation of oxalate in the bloodstream, with potentially devastating complications affecting organs beyond the kidneys (e.g., bone fractures, vision loss, heart problems) due to the ensuing deposition of calcium oxalate crystals throughout the body [2, 4, 6]. PH1 is associated with early mortality due to ESKD and complications of systemic oxalosis (i.e., oxalate deposition throughout the body, beyond the kidneys) [7].

PH1 has an estimated prevalence of 1 to 3 cases per million according to epidemiology data from Europe. Prevalence may be elevated in populations with increased rates of consanguinity (i.e., birth of children to parents who are related to one another) and populations with pathogenic founder mutations (i.e., populations in which many individuals are descended from a single common ancestor who carried a PH1-causing mutation) [1, 3, 8].

Symptoms of PH1 typically first arise around 4 to 5 years of age [9, 10]. Because of the rarity of PH1 and the potential non-specificity of disease symptoms, diagnostic delay is common; median time from the first appearance of symptoms to diagnosis exceeds 5 years [11, 12]. Up to 70% of adults and more than 40% of pediatric patients with PH1 have already reached ESKD by the time of diagnosis [1, 7, 10, 11, 13].

PH1 carries a heavy acute symptom burden associated with kidney stones: excretion of blood in urine, difficult or painful urination, abdominal pain, urinary tract blockage, and recurrent urinary tract infections [2]. High rates of symptoms potentially associated with impacts of PH1 beyond the kidneys and urinary tract are also reported [8]. These symptoms are in addition to the potential CKD symptoms, such as impaired cognitive and physical abilities, sleep disturbance, and pain, that can occur in association with PH1 [14]. Patients with PH1 in whom CKD has progressed to ESKD and/or who require dialysis may experience a further increase in symptom burden due to ESKD and dialysis (e.g., fatigue, difficulty sleeping, bone or joint pain, sexual dysfunction, itching, muscle cramps, dry mouth) in addition to the potential future burden of liver-kidney transplantation, which is associated with significant risks of death and other complications [15–17].

Historically, PH1 management has mainly involved treatment with high-dose vitamin B_6_ and/or other supportive measures (increased fluid intake, use of oral citrate supplements) aimed at creating unfavorable conditions for excess oxalate to combine with calcium to form calcium oxalate crystals, with these measures followed by dialysis and ultimately transplantation of the liver (to remove the source of excess oxalate production) and kidneys (to replace kidney function lost to oxalate-induced kidney damage) once late-stage CKD is reached [1, 3, 4, 15, 18, 19]. In 2020, lumasiran became the first PH1 treatment approved by the US Food and Drug Administration (FDA) and the European Medicines Agency (EMA) [3]. Adults and pediatric patients receiving lumasiran experienced substantially reduced oxalate levels across all disease severity levels, including advanced cases requiring dialysis, in three Phase 3 clinical trials; the most common adverse events related to lumasiran were mild, short-lived injection-site reactions [19–21]. Nedosiran, which in the Phase 3 PHYOX 2 trial demonstrated a significantly greater reduction in urinary oxalate versus placebo from Day 90 to Day 180 [22], has since become the second FDA-approved PH1 treatment; its indication is limited to patients 9 years of age and older with relatively preserved kidney function. No other approved treatments are available. Other treatments under study include a pharmaceutical formulation of the bacterium Oxalobacter formigenes [23], stiripentol, CHK-336, betaine, and oral enzyme therapies [3, 15].

Qualitative research methods, including semi-structured interviews featuring both open-ended and more targeted questions, are often recommended by the FDA to gain insights into patients’ experiences and elicit the factors important to patients in the context of new therapy development [24, 25]. In addition, understanding how patients experience disease burden is critical for informing care decisions that best meet patients’ needs. This is particularly important in PH1, given that care is evolving, new treatments are emerging, and treatment decisions are becoming more complex.

Only one study has qualitatively examined patient and caregiver experiences in PH, spanning PH1, PH2, and PH3 [26]. We therefore aimed to conduct a study to describe patients’ and caregivers’ self-reported experiences in the specific setting of PH1, with a unique focus on the diagnostic journey and on differences in experience by disease stage and age. Regarding disease experience, we aimed to gather information on the impacts of PH1 on patients and their caregivers specific to activities of daily living, social activities, physical and emotional well-being, and work/school; explore patients’ and caregivers’ experiences and expectations relating to management of PH1; and describe the support and resources that these patients and caregivers access.

## Methods

### Study design

In this observational study, experienced qualitative research staff conducted in-depth telephone interviews with patients diagnosed with PH1 or their caregivers. To ensure consistent and accurate data capture, each 60-min semi-structured interview was recorded and facilitated by 2 staff members: an interviewer led the discussion, and the second staff member captured detailed notes and ensured adherence to the interview guide. Participants were recruited through qualitative research partners Global Perspectives and Rare Patient Voice, and an institutional review board determined exemption status. Because the study took place during the COVID-19 pandemic (17 June 2020 to 23 February 2021), the interviews also addressed the impact of COVID-19 overall, and on living with PH1 specifically.

### Study population

A screening tool confirmed participant eligibility and informed consent was provided. Study-eligible individuals were patients aged 12 years and older and caregivers of children aged 6 to 17 years with a genetically confirmed diagnosis of PH1. Participants were required to provide informed consent/assent/parental permission (as applicable) and be able to read and speak English. The study protocol specified a recruitment target of 28 patients.

### Study variables and interview concepts

Demographic data and other baseline characteristics were collected at screening. The interview discussion guide elicited descriptions of early symptoms that prompted efforts to seek medical care, and an overall summary (from the patient or caregiver perspective, as applicable) of symptoms experienced throughout the disease course, impacts related to those symptoms, experiences with the management of PH1, treatment expectations, support sources, resources accessed, and future outlook.

### Analysis

A constant comparative analysis method was employed to reveal dominant trends in interview data and thus identify themes or patterns in participants’ descriptions of their experiences and perceptions [27]. Specifically, interview transcripts and interviewer field notes were used to identify dominant trends within each recorded interview, and then each interview’s findings were compared with those of the other interviews to identify themes or patterns recurring across the interview sample in terms of participants’ descriptions. According to the calculation method of Fugard and Potts [28], the target study cohort size of 28 patients would provide 90% power to detect any theme of interest in at least 1 interview if the true prevalence of the patient experience captured by that theme was between 5 and 10% in the underlying PH1 population represented by the study cohort.

For analysis of baseline quantitative and categorical data, descriptive statistics were computed, quality checked, and summarized.

## Results

### Study population

In total, 16 patients with PH1 (10 adults, 6 adolescents) and 12 caregivers (of children aged 6–16 years with PH1) were interviewed. Interviewees’ baseline demographics and disease characteristics are presented in Table 1.

**Table 1:** Interview participant and care recipient demographic characteristics

Characteristics	Patient Interviewees (Adults and Adolescents) *N* = 16	Caregiver Interviewees on Behalf of Care Recipients (Children Aged 6–16 Years) *N* = 12	Total *N* = 28
**Sex of interview participant,**^**a**^** n (%)**			
Male	8 (50.0)	4 (33.3)	12 (42.9)
Female	8 (50.0)	8 (66.7)	16 (57.1)
**Sex of patient**^**b**^ **with PH1 (interview participant in the case of patient interviewees; care recipient in the case of caregiver interviewees), n (%)**			
Male	8 (50.0)	6 (50.0)	14 (50.0)
Female	8 (50.0)	6 (50.0)	14 (50.0)
**Patient**^**b**^** age, mean (range), y**	25.8 (16–51)	10.1 (6–16)	19.1 (6–51)
**Patient**^**b**^** kidney function/status, n (%)**			
Normal	6 (37.5)	7 (58.3)	13 (46.4)
Abnormal (without dialysis or transplantation)	3 (18.8)	2 (16.7)	5 (17.9)
On dialysis	2 (12.5)^c^	2 (16.7)	4 (14.3)
Post-kidney and liver transplantation	5 (31.3)	1 (8.3)	6 (21.4)
**Patient**^**b**^** race/ethnicity, n (%)**			
White	15 (93.8)	9 (75.0)	24 (85.7)
Black	1 (6.3)	1 (8.3)	2 (7.1)
Mixed	0 (0.0)	2 (16.7)	2 (7.1)
Hispanic or Latino	1 (6.3)	1 (8.3)	2 (7.1)
**Interview participant education,**^**a**^** n (%)**			
Less than high school	6 (37.5)	0 (0.0)	6 (21.4)
High school or equivalent	0 (0.0)	2 (16.7)	2 (7.1)
Associate or technical degree	0 (0.0)	1 (8.3)	1 (3.6)
Some college but no degree	5 (31.3)	1 (8.3)	6 (21.4)
College degree	4 (25.0)	7 (58.3)	11 (39.3)
Graduate or professional degree	1 (6.3)	1 (8.3)	2 (7.1)

^a^Interview participants are patients > 16 years of age and care providers for patients 6–16 years of age

^b^In the case of caregiver interviewees, “patient” refers to the patient with PH1 who is being cared for by the interviewee

^c^Includes 1 participant who reported previous experience with dialysis but, due to improvements in kidney function, has now been able to stop dialysis treatments. For the purposes of the report, this participant is included in the dialysis treatment group

### Pathway to diagnosis

Average age at diagnosis of PH1 was 8.8 years (range, pre-birth to 32 years), and average time from the first appearance of symptoms to PH1 diagnosis was 1.8 years (range, 2 months to 10 years). Interviewees reported that symptoms that led to diagnosis included kidney stones (96%), pain associated with kidney stones (84%), tiredness/fatigue (48%), fever/chills during kidney stone episodes (40%), painful urination due to kidney stones (40%), frequent urge to urinate (40%), and bed-wetting (36%).

*“It was… since I was 9 months old, my kidneys failed. Then I had a kidney transplant when I was about 1 year old. I was misdiagnosed when I had the kidney transplant, and then they found out when I was 6 years old*.” (adult patient)

“*[O]ne of the issues initially was bed wetting and initially his pediatrician chalked that up to the communication between the brain and the bladder, the age of development. [They thought it] would eventually go away.*” (caregiver)

Barriers to diagnosis noted by interviewees included problems with insurance coverage for PH1 genetic testing, lack of knowledge of PH1 by healthcare providers, time to referral for a nephrologist, dismissal of symptoms by healthcare providers, and time to receive genetic testing results:

“*The doctors, my primary care and then my kidney doctor that I had, they had never even heard of it [PH1].”* (adult patient)

### PH1 symptoms following diagnosis

Most patients (71%) were reported to have 1 to 4 ongoing PH1 symptoms that continued following diagnosis, the most common being kidney dysfunction (Table 2). Patients who experienced ongoing symptoms following diagnosis were reported to have kidney, skeletal (with joint pain and swelling), heart, skin, and/or eye symptoms, as well as other nonspecific constitutional symptoms. When asked to report the most bothersome symptoms of PH1, interviewees most frequently noted pain associated with kidney stones (*N* = 11) and fatigue (*N* = 6) (Table 3).

“*Kidney stones for sure. Just the pain. It was absolutely miserable. Sometimes I was mildly in pain, and sometimes I wanted to lie in the fetal position and cry*.” (adult patient)

“*[T]he fatigue is a concern to me. Because he shouldn’t be that tired*.” (caregiver)

**Table 2:**
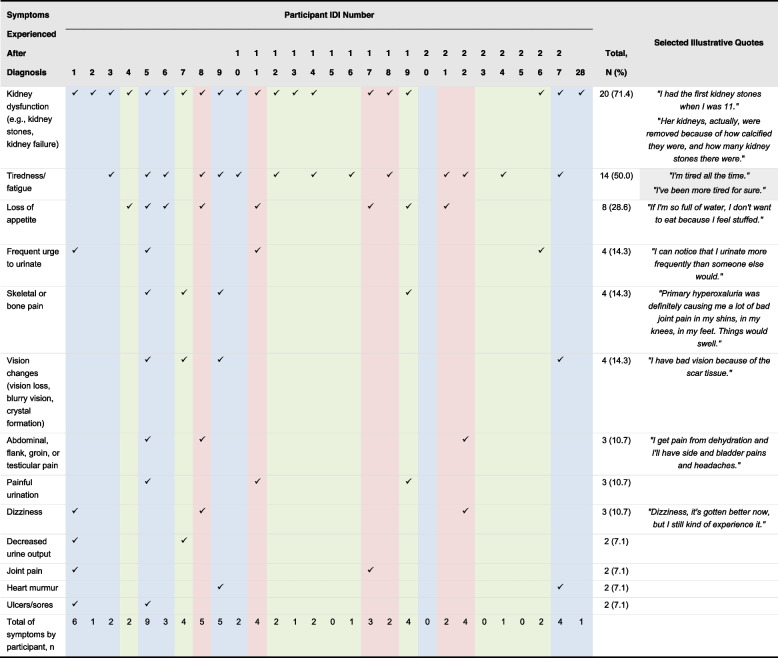
Symptoms of PH1 reported by patients after diagnosis

Symptoms experienced by 1 patient only included swelling in fingers, inability to focus, headaches, feeling generally ill, skin discomfort/pain, palpitations, shortness of breath, fractures, and nausea

*IDI* In-depth interview, *PH1* Primary hyperoxaluria type 1

Blue columns = adult patient participants; red columns = adolescent patient participants; green columns = care provider participants

**Table 3:** Most bothersome symptom

Symptom	Patients (Adult and Adolescent) *N* = 14	Care Recipients ^a^ *N* = 10	Total *N* = 24^b^
Pain of kidney stones	7	4	11
Fatigue	5	1	6
Other physical pain (e.g., stomach pain, leg pain, and headache)	0	3	3
Bed-wetting	0	2	2
Frequent urination	1	2	3
Painful urination	0	1	1
Skin problems (e.g., blisters)	1	0	1
Weakened immune system	0	1	1

^a^Most bothersome symptoms for care recipients reported by caregivers. Interviewees could indicate more than 1 “most bothersome” symptom

^b^Four patients did not provide a response

### Impacts of PH1

Adult patient interviewees more frequently reported impacts of PH1 in terms of physical activity/mobility (50%) and depression (40%), whereas adolescent patient interviewees reported health-related worries (83%) and impacts on social functioning (83%) (Table 4). In addition, 10 of the 12 caregiver interviewees reported 1 or more impacts on their own lives; impacts on caregivers included disturbed sleep, worry and fear for their children's future, missing work, inability to care for self (e.g., hygiene/grooming, dealing with medical care, exercise), and impacts on daily activities (e.g., house cleaning).

**Table 4:**
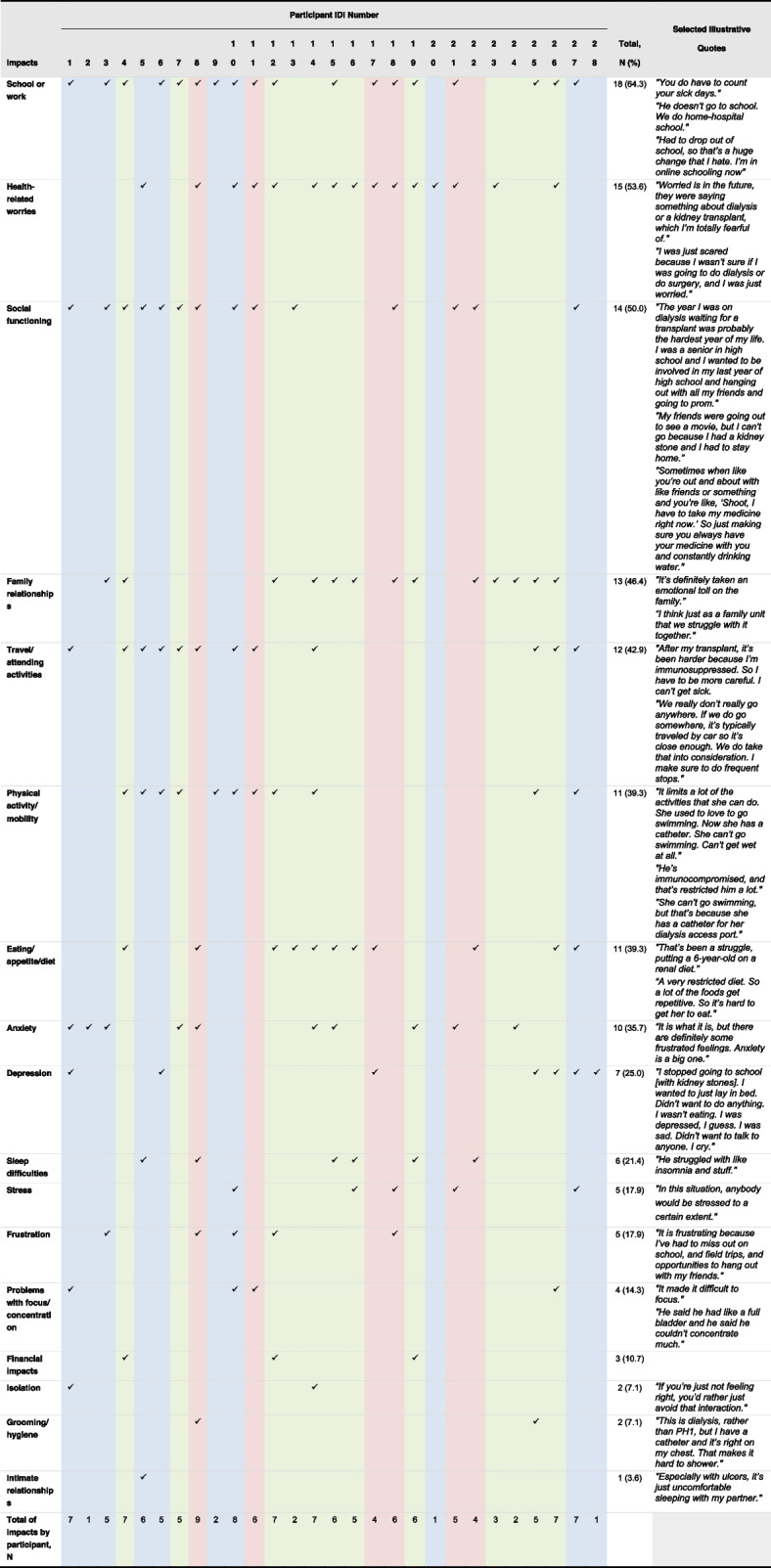
Impacts of PH1 reported by patients

*IDI* In-depth interview, *PH1* Primary hyperoxaluria type 1

Blue columns = adult patient participants; red columns = adolescent patient participants; green columns = caregiver participants

Interviewees were also asked about how the impact of PH1 evolved over time. In total, 10 interviewees said some impacts have become more challenging with time, such as disease management requirements (e.g., taking medication on time, drinking sufficient water) (*N* = 3), scheduling doctor visits and routine testing (*N* = 2), waiting for a transplantation (*N* = 2), fear of future need for dialysis and/or transplantation (*N* = 1), working with insurance companies (*N* = 1), and problems with catheter access points (*N* = 1).

*“I have to be more diligent… about taking my medicine, where before transplant, it was like, okay, if I miss a dose it’s not the end of the world. Where now, if I miss a dose of my antirejection, it is a big deal.”* (adult patient)

Study participants were also asked to describe the most bothersome and/or most difficult-to-manage impacts of PH1. Impacts related to disease management requirements (e.g., diet, fluid intake, dialysis) (*N* = 7) and social life impacts (*N* = 7) were deemed the most bothersome by both patients and caregivers (Table 5). Patients and caregivers reported that the impacts of disease management requirements (*N* = 5), disease impacts on social life (*N* = 3), and impacts on emotional/mental well-being (*N* = 3) were the most difficult-to-manage impacts of PH1 (Table 6).

**Table 5:** Most bothersome impact

Impact	Patient (Adult and Adolescent) *N* = 11	Care Recipients ^a^ *N* = 10	Total ^b^ *N* = 21	Selected Illustrative Quotes
Treatment requirements (e.g., diet, fluid intake, dialysis)	1	6	7	*"I would think it's definitely, even though he's okay with the lab work, the lab work, having to watch his diet."*
Social life	6	1	7	*"I think socializing, it's not that I can't talk to anybody. It's that it's just, it's a bit uncomfortable."* *"I would say the social aspect."* *"I think missing out with my friends."*
Emotional/mental impact	3	1	4	*"Thinking about going back and having dialysis."*
Ability to attend school	1	0	1	
Doctor visits	0	1	1	
Dressing (i.e., need to wear liners due to frequent urination)	0	1	1	

^a^Most bothersome impacts for care recipients reported by caregivers

^b^Seven patients did not provide a response to this question

**Table 6:** Most difficult to manage impact

Impact	Patient (Adult and Adolescent) *N* = 9	Care Recipients ^a^ *N* = 6	Total ^b^ *N* = 15	Selected Illustrative Quotes
Treatment requirements (e.g., diet, flud intake, dialysis)	3	2	5	*"I just think that she struggles sometimes drinking the water.”* *“Just taking medicine on time [is] tricky."*
Social life	3	0	3	*"Just want to still have a social life or go out."*
Emotional/mental impact	1	2	3	
Physical limitations (e.g., swimming)	0	1	1	
Doctor visits	0	1	1	
Dressing (i.e., need to wear liners due to frequent urination)	0	1	1	
Difficulty with future pregnancy	1	0	1	

^a^ Most difficult to manage impacts for care recipients reported by caregivers

^b^Thirteen patients did not provide a response to this question

### Experiences related to the management of PH1

Study participants were asked about their experiences related to the management of PH1, including COVID-19–related impacts on these experiences. Regarding specific disease management options, 10 patients were reported to have a history of transplant or dialysis: *N* = 6 (as reported by 5 patient interviewees and 1 caregiver interviewee) had undergone kidney and liver transplantation, and *N* = 4 (as reported by 2 patient and 2 caregiver interviewees) were on dialysis at the time of the interview, or sometime previously.

Among patients with current (*N* = 3) or past (*N* = 1) dialysis, schedules ranged from 3 to 6 sessions per week lasting 3 to 4.5 h each. Patients described symptoms of fatigue, headache, and dizziness following dialysis sessions. Study participants also described practical limitations caused by the need to keep catheter access sites dry, and significant time commitments for sessions themselves and for travel to the dialysis site. Caregiver interviewees with children on dialysis described the impact of dialysis schedules on their lives, routines, and families.

*"Day starts off with dialysis; get home in the afternoon. So because of her really full schedule with dialysis, she’s had to switch to homebound [instructional services] for school. So a teacher comes 2 days a week for only 2 hours at a time. And before this, she was enrolled in a regular classroom with 20 other kids. And now it’s just her and the teacher at the dining room table. I know there’s only so much they can do with homeschool, but it’s definitely a reduced curriculum. And so sometimes we feel she’s missing out*." (caregiver)

COVID-19–related impacts on the experience of dialysis were described as well; the study findings suggest that the pandemic exacerbates the challenges of dialysis by increasing the duration and complexity of visits, making it more difficult for caregivers to accompany and support young patients during dialysis sessions.

*“My child is on dialysis, and she goes 6 days a week for treatments, so the process of getting checked-in and ready for dialysis is a lot different than it was a few months ago with two temperature checks. It’s like there’s a lot of checkpoints. When we first get there, we get our temperature checked. We get asked a whole bunch of questions. When we get to the renal floor, it’s kind of the same routine.”* (caregiver)

Short-term impacts described in association with kidney/liver transplantation (*N* = 6) included pain, physical limitations, missed school, and inability to spend time with friends in the period immediately following the procedure. Over the longer term, transplantation was described as having positive impacts on patients’ lives, including increased energy, a feeling of recovered health, the ability to stop dialysis, and cessation of kidney stones. Accompanying these positive impacts, however, were certain long-term concerns, including concerns over the need for “antirejection medications” (and the need for tight adherence to the recommended dosing schedule for these medications) following transplantation, as well as concerns over the potentially harmful effects of immune system suppression caused by these antirejection medications.

*“Just taking medicine on time [is] tricky and making sure I drink a lot of water can be tricky.”* (adult patient)

Some interviewees also described the potential need for and emotional and physical impact of additional transplantations (e.g., following failure of a transplanted organ) in the future.

"*The first month or so [post-transplantation], it’s just recovering. That’s always different. After that, I had way more energy, and it was good… I know someday I’ll have to get a transplant again, hopefully not. That’d be awesome. Other than that, I know I’ll have to take medicine forever, but I feel like I have it under control. I think I can manage it*." (adult patient)

Among patients with abnormal kidney function who had not undergone dialysis/transplantation (*N* = 5; as reported by 3 patient interviewees and 2 caregiver interviewees), disease management involved high fluid intake/hyperhydration for all 5. Similarly, among those with normal kidney function (*N* = 13; as reported by 6 patient interviewees and 7 caregiver interviewees), a regimen of high fluid intake/hyperhydration was reported for all 13. Other disease management measures noted by these 13 interviewees included the use of vitamins (with vitamin B_6_ specifically reported for 7 patients), a low-sodium diet, and prescription medications to help pass kidney stones.

“*Every night I set out an amount of bottles. I have an app on my phone that can track my water*.” (adolescent patient)

“*She has one large, I want to say it’s a little over half a gallon water bottle that she fills up and she drinks that. Then she has her, sports drink that she tries to drink. She takes her vitamin drops, and she’ll put that in her water too and that helps. I know that she tries to go above. The doctor was recommending the eight to nine glasses, and I know that she goes…at least one to two bottles over.”* (caregiver)

### Expectations of treatment

The majority of interviewees (75%) indicated that a treatment that could lower laboratory measures of oxalate burden would provide a meaningful benefit.

“*I think if a medication could help lower the oxalate somehow, whether it works by replacing that enzyme in the liver or if it helps your kidneys get rid of it or whatever. If it somehow helps lower oxalate, even if it's just a little bit, I think that that would be a wonderful change*.” (adult patient)

Half of all interviewees also reported they would value treatments that reduced physical symptoms, for example, by reducing the frequency of kidney stones. Table 7 presents other factors of importance in PH1 medication development that were identified by the respondents.

**Table 7:** Factors of importance in PH1 medication development

Factor	Total	Selected Illustrative Quotes
Improve lab results (e.g., decreased oxalate)	*N* = 21; 75%	“*But if it makes me feel better or makes those values [oxalate] decrease even* *more, then…I’d be willing to try something else.*”
Physical symptoms: • Reduce frequency and severity of kidney stones • Improve fatigue/energy levels • Decrease frequency of urination • Reduce/eliminate ulcers	*N* = 14; 50%	“*The thought of not having to even go through a kidney stone, that would be incredible*." “*If I got less kidney stones.*” *“We’re definitely interested for any medications that can make her, help her lead a normal life without having, to have such frequent urination…”* “*I would say reduce as many symptoms [ulcers] as possible*.”
Prevent need for dialysis or kidney/liver transplantations	*N* = 6; 21%	*"There is no cure, but I think if you can lessen the chances of having to be put on dialysis or get a transplant, I think that that is a very successful medication that I would love to have had."*
Reduce need for increased fluid intake	*N* = 5; 18%	"*If there was any way that it could reduce the amount of water they have to drink. Water is a four-letter word in our house, and it’s not fun. And trying to get a child to drink that much water every single day is so hard."*
Allow for more flexibility in diet	*N* = 3; 11%	
Reduce emotional impacts	*N* = 3; 11%	*"I think those emotional impacts would come as a result of the other symptoms being lessened, let’s say fatigue or those aches and pains which correlate in their own way emotionally as a result of how extreme is the fatigue, how extreme is the pain."*

### Resources and looking ahead

Adult patients’ and caregivers’ primary sources of support include their doctor (*N* = 3), partner (*N* = 3), family/friends (*N* = 6), Facebook support groups (*N* = 1), church (*N* = 1), and therapist (*N* = 1). Other sources of support for patients and caregivers included online resources, nutritionists/dieticians, support groups through local hospitals, and child psychologists. Fourteen participants reported having connections with other individuals/families affected by PH1.

Participants reported that support could be improved through enhanced training and awareness among medical professionals, increased access to support groups, development of targeted disease information/resources based on patient characteristics (e.g., gender, age), more information on financial resources and dealing with insurance providers, and more drug development/clinical trials to provide other treatment options.

## Discussion

In broad agreement with findings from the study by Lawrence and Wattenberg across all types of PH [26], the current study found that PH1 substantially impacts physical and emotional health [26]. The current study also expands on such previous work by focusing on the experience of patients with PH1 specifically, and by gathering data on the diagnostic journey and elucidating differences in experiences by disease stage and age.

The results of the current study corroborate the well documented role of kidney stones and related symptoms in triggering diagnosis of PH1 [10], as well as the common occurrence of delays in diagnosis following onset of clinical manifestations [10]. From patients’ and caregivers’ perspectives, issues with insurance coverage for genetic testing, limited awareness of PH1 by healthcare providers, and long wait times for referral to a nephrologist were key factors contributing to these delays.

Post diagnosis, nearly all patients experienced symptoms of PH1. Kidney-related symptoms were common, but a number of patients also reported symptoms beyond the kidneys, such as fatigue, bone pain, and vision changes. The practical impacts of these symptoms, and of the disease more broadly, appeared to differ according to the age of the patient. Adolescents frequently reported psychological and/or social impacts (worry and impaired social functioning), whereas adults reported impacts on physical functioning and emotional well-being (depression). The practical impacts of PH1 extended to caregivers, who reported issues ranging from sleep disturbance to impairment in emotional well-being, occupational outcomes, and self-care/activities of daily living.

Regarding PH1 management, there is a heavy burden imposed by the need for dialysis in late-stage PH1. Interviewees discussed how dialysis is all-consuming due to the required travel and chair time, leading to social, educational, and occupational limitations. Interviewees also reported that catheters impose practical restrictions (e.g., the need to keep the access site dry complicates daily activities such as showering). These impacts are in addition to the impacts of the dialysis procedure itself, which often produces side effects such as fatigue, headache, and dizziness. Finally, interviewees noted that the challenges of dialysis are exacerbated by the COVID-19 pandemic, which further increases the duration and complexity of visits and makes it more difficult for caregivers to accompany and support young patients.

Our study provides unique insight into the treatment benefits desired by patients and caregivers. Improvement in laboratory measures of oxalate burden is highly valued by study participants. Oxalate is recognized as the starting point from which clinical burden originates, and patients and caregivers communicated that improvement in indicators of oxalate burden would thus have an important impact on their emotional well-being. The perceived value of reductions in oxalate burden is strongly linked to participants' desire for a treatment that halts disease progression and prevents the need for dialysis. Reduction in physical symptoms (e.g., kidney stone events, skin ulcers, and frequent urination) is the next most highly valued treatment benefit, representing significant alleviation of the physical burden of PH1.

We used a robust qualitative methodology to describe the experience of patients living with PH1 and their caregivers. Information was collected directly from patients and caregivers, and interviews were recorded, transcribed verbatim, and analyzed by highly experienced qualitative researchers. To facilitate comparability across interviews with different study participants, a comprehensive semi-structured guide was used in conducting all interviews. The use of a robust, systematic analysis method to extract meaning from unstructured qualitative data provided key insights and allowed meaningful interpretation of the study findings.

This study was limited by the relatively small sample size, as is frequently the case with rare diseases. In addition, recall challenges are a limitation of studies that involve self-reporting. To help limit bias and reduce the interviewer’s influence on response, interviews centered on open-ended questions. The study is also subject to the inherent limitations of qualitative analysis, for example, inability to draw conclusions about statistical relevance and limited generalizability of findings. Finally, it should be noted that study interviews were conducted between June 2020 and February 2021, with most completed before FDA approval of lumasiran in November 2020, and the study protocol excluded patients who had received an experimental therapy at any point in the 90 days before eligibility screening. Therefore, although patients were not asked whether they had experience with lumasiran or an experimental PH1 therapy, the findings reported here can be viewed as being reflective of patients’ experience with PH1 in the context of older, historical disease management interventions rather than newer therapies.

Overall, the findings of this study afford clinicians and decision-makers insights into the humanistic burden of PH1 in terms of physical, emotional, and practical impacts as described by affected individuals and their caregivers. These results may offer healthcare providers an increased understanding and awareness of PH1 and the burden experienced by both patients and caregivers. The study findings, combined with existing data on the burden of PH1, may also help inform clinical decision-making related to diagnosis and treatment and ensure patients’ needs are considered in the context of PH1 management [24].

## Data Availability

The authors will share deidentified screening, inclusion, and exclusion data upon reasonable request, which may be directed to Carla Romano (demuromercon@rti.org). The authors do not have permission to share other, transcript-associated data given that the patients have a rare disease and are potentially identifiable.

## Abbreviations

CKD: Chronic kidney disease
ESKD: End-stage kidney disease
FDA: Food and Drug Administration
PH: Primary hyperoxalurias
PH1: Primary hyperoxaluria type 1
